# Recent Advances in Synthetic Biology Approaches to Optimize Production of Bioactive Natural Products in Actinobacteria

**DOI:** 10.3389/fmicb.2019.02467

**Published:** 2019-11-05

**Authors:** Lei Li, Xiaocao Liu, Weihong Jiang, Yinhua Lu

**Affiliations:** ^1^Key Laboratory of Synthetic Biology, CAS Center for Excellence in Molecular Plant Sciences, Institute of Plant Physiology and Ecology, Chinese Academy of Sciences, Shanghai, China; ^2^School of Life Sciences, Henan University, Kaifeng, China; ^3^Jiangsu National Synergetic Innovation Center for Advanced Materials, SICAM, Nanjing, China; ^4^College of Life Sciences, Shanghai Normal University, Shanghai, China

**Keywords:** actinobacteria, natural product, synthetic biology, dynamic regulation, BGC amplification, pathway refactoring, genome-minimized host

## Abstract

Actinobacteria represent one of the most fertile sources for the discovery and development of natural products (NPs) with medicinal and industrial importance. However, production titers of actinobacterial NPs are usually low and require optimization for compound characterization and/or industrial production. In recent years, a wide variety of novel enabling technologies for engineering actinobacteria have been developed, which have greatly facilitated the optimization of NPs biosynthesis. In this review, we summarize the recent advances of synthetic biology approaches for overproducing desired drugs, as well as for the discovery of novel NPs in actinobacteria, including dynamic metabolic regulation based on metabolite-responsive promoters or biosensors, multi-copy chromosomal integration of target biosynthetic gene clusters (BGCs), promoter engineering-mediated rational BGC refactoring, and construction of genome-minimized *Streptomyces* hosts. Integrated with metabolic engineering strategies developed previously, these novel enabling technologies promise to facilitate industrial strain improvement process and genome mining studies for years to come.

## Introduction

As a kind of Gram-positive bacteria with high GC content, actinobacteria undergo complex morphological differentiation and secondary metabolism processes (Barka et al., 2016). Actinobacteria, particularly the *Streptomyces* genus, have been recognized as the main sources for microbial bioactive natural products (NPs), such as antibiotics, chemotherapeutics, immunosuppressants and anthelmintics, which make important contributions to health care and crop protection (Jensen et al., 2015; Genilloud, 2017; Nepal and Wang, 2019). With the rapid advances in genome sequencing and genome mining methods (Ziemert et al., 2016; Blin et al., 2019), a wealth of hidden NP biosynthetic gene clusters (BGCs) have been revealed by specialized software (i.e., antiSMASH) and are regarded as an untapped treasure trove for the discovery of novel bioactive compounds (Rutledge and Challis, 2015; Niu, 2018). For instance, a single *Streptomyces* genome usually harbors around 30 NP BGCs, approximately 10-fold more than previously identified by bioactivity screening (Rutledge and Challis, 2015). However, the majority of NP BGCs in actinobacteria are silent or cryptic under laboratory culture conditions, and must be activated for the isolation and characterization of unknown compounds (Rutledge and Challis, 2015; Zarins-Tutt et al., 2016). Furthermore, production titers of many available actinomycete-derived drugs are still low for the economically viable industrial bioprocess. Construction of highly efficient microbial cell factories becomes increasingly critical for commercial application of desired NPs (Kim et al., 2016; Zhang et al., 2016).

With the advents of metabolic engineering and synthetic biology, genetic engineering of actinobacteria could address several major challenges associated with NP discovery, development and large-scale manufacturing. In the last three decades, a variety of genetic engineering strategies have been developed for strain development, including precursor engineering, BGC amplification, deletion of competing pathways, engineering of translational/transcriptional machineries as well as manipulation of pleiotropic/pathway-specific regulators (Baltz, 2016; Kim et al., 2016; Palazzotto et al., 2019). Interested readers are referred to the detailed reviews of systems biotechnology of actinobacteria (Weber et al., 2015; Baltz, 2016; Kim et al., 2016; Zhang et al., 2016; Palazzotto et al., 2019). It is worth noting that with breakthroughs in CRISPR-based genome editing methods, a series of novel enabling technologies have been developed, greatly facilitating the engineering of actinomycetal genomes (i.e., deletion, insertion, replacement and point mutation) as well as NP BGCs (i.e., cloning, editing, deletion and amplification) (Li et al., 2017a; Tong et al., 2018; Alberti and Corre, 2019). In this review, we briefly summarize the most recent synthetic biology approaches and discuss how these technologies enable the generation of microbial cell factories and the discovery of novel therapeutic drug leads. These include dynamic regulation based on metabolite-responsive elements, multiplex site-specific recombination (SSR) system-mediated BGC amplification (MSGE), systematical and rational BGC refactoring, as well as construction of genome-minimized *Streptomyces* hosts for NP overproduction and discovery. Together with traditional metabolic engineering strategies (Weber et al., 2015; Baltz, 2016), we believe that these newly developed tools will be widely applicable for actinobacteria, providing general strategies for (meta)genome mining-based novel NP discovery as well as for the overproduction of commercially important NPs.

## Dynamic Pathway Regulation Based on Metabolite-Responsive Promoters or Regulators

Dynamic metabolic regulation has proved to be an effective strategy to improve production titers of target compounds by balancing bacterial growth and biosynthesis of specific metabolites (Zhang et al., 2012; Dahl et al., 2013; Gupta et al., 2017). Generally, three different approaches – quorum sensing systems, metabolite-responsive promoters and protein/RNA-based biosensors – are used for autonomous control of metabolic pathway flux (Zhang et al., 2015; Polkade et al., 2016; Tan and Prather, 2017). These new concepts were initially developed in *Escherichia coli* or *Saccharomyces cerevisiae*, and the latter two have been extended to actinobacteria for the optimization of antibiotic biosynthetic pathways.

### Metabolite-Responsive Promoters

**FIGURE 1:**
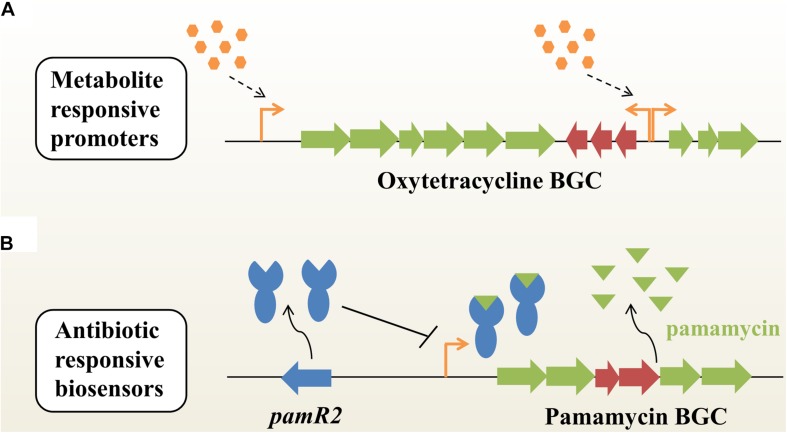
Dynamic pathway regulation balances flux between bacterial growth and antibiotic biosynthesis in actinobacteria. **(A)** Autoregulated fine-tuning of the expression of an antibiotic (e.g., oxytetracycline) BGC based on metabolite-responsive native promoters without specific transcription factors or additional inducers. BGC, biosynthetic gene cluster. **(B)** Antibiotic-responsive biosensors. This strategy is engineered from cluster-situated regulators (e.g., PamR2) that bind to their associated promoters upon interaction with the corresponding secondary metabolites (e.g., pamamycins). Placing antibiotic biosynthetic genes under such promoters enables antibiotic dependent gene expression.

In the last three decades, a large variety of metabolic engineering strategies have been developed to optimize production of secondary metabolites in microbes. However, few approaches could enable the coordination between bacterial growth and biosynthesis of target compounds. Recently, Li et al. (2018) employed time-course transcriptome analysis to identify a series of antibiotic-responsible promoters with a transcription profile similar to the inducible promoters, when under the optimal conditions (Figure 1A). These dynamic responsive promoters could be used to efficiently optimize the expression of native actinorhodin and heterogeneous oxytetracycline (OTC) BGCs in *Streptomyces coelicolor*, subsequently improving the production titers of ACT and OTC by 1.3- and 9.1-fold, respectively, compared with constitutive promoters (Li et al., 2018). Xu et al. (2012) reported a metabolite-responsive promoter, which controlled the transcription of *actAB* encoding an antibiotic exporter in *S. coelicolor*. They found that the antibiotic ACT and its biosynthetic intermediates [e.g., (S)-DNPA] could relieve the repression of *actAB* by binding the transcriptional regulator ActR. That means that the *actAB* promoter could indirectly respond to intermediates or end-products, thus synergistically regulating ACT biosynthesis and export. The metabolite-responsible promoter based strategy will achieve an autonomous induction of pathway regulation and provide a universal route for titer improvements of desired NPs in actinobacteria.

### Natural Product-Specific Biosensors

Genetically encoded biosensors are a kind of important synthetic biology tool for real-time detection of intracellular metabolites for specific readouts (Zhang et al., 2015). A typical biosensor is composed of three modules: (i) a signal input module, such as transcription factors (TFs) or riboswitches; (ii) a regulatory module, such as TF-dependent promoters; (iii) a signal output module, such as reporter genes (Mahr and Frunzke, 2016; Lim et al., 2018). Until now, three major categories of biosensors - fluorescence resonance energy transfer (FRET)-based sensors, TF-based sensors and riboswitches – have been widely used in metabolic engineering of microbial hosts (Zhang et al., 2015; Lim et al., 2018). As a kind of natural sensory protein, a classical TF has evolved to contain a ligand-binding domain for responding to environmental changes and a DNA-binding domain for regulating gene expression. Interestingly, many NP BGCs encode cluster-situated regulators (CSRs) in actinobacteria, such as TetR-like regulators and *Streptomyces* antibiotic regulatory proteins (SARPs; Liu et al., 2013; Romero-Rodriguez et al., 2015). These CSRs can be used to dynamically detect developmental state, population density or other environmental changes, and thus determine the onset and production levels of secondary metabolites in actinobacteria. Recently, Rebets et al. (2018) developed a new antibiotic-specific whole-cell biosensor based on a TetR-like repressor for the development of antibiotic-overproducing strains (Figure 1B). Briefly, the highly active macrodiolide antibiotic pamamycins BGC encodes a transporter PamW and a corresponding transcriptional repressor PamR2, which can be deactivated by binding to pamamycins (Rebets et al., 2015). Generally, PamW expression is controlled by PamR2 at low level of pamamycins. However, at very high levels of pamamycins, the native PamR2-based biosensors will reach the detection limit and cannot effectively regulate PamW expression (Rebets et al., 2018). The *pamW* promoter was directly used to control expression of the kanamycin resistance gene, thus generating the resistance-based G0 whole-cell biosensor system. After UV-induced mutagenesis, strains resistant to a high-concentration of kanamycin showed a significant increase in pamamycins production (up to 15–16 mg/L). Of note is the fact that the native pamamycins-responsive biosensor showed limited operating and dynamic ranges for further applications. To overcome this obstacle, the G1 pamamycins biosensor was further developed by combining different promoters, varying the number and position of operators, as well as using diverse reporter genes. Using the new biosensor with higher sensitivity, three mutated strains were obtained, which could produce up to 30 mg/L of pamamycins. Furthermore, to overcome the low detection limit of the G1 biosensor, the binding affinity of PamR2-pamamycins was also efficiently decreased by designing a panel of PamR2 mutations. As expected, the G2 biosensor showed a better operating dynamic range than that of the G1 biosensor (Rebets et al., 2018). In fact, at least 17% of NP BGCs encode TetR-like regulators and putative transporters simultaneously, which provides possibilities for the development of diverse antibiotic-responsive biosensors (Rebets et al., 2018). With the aid of a metabolite-responsive biosensor system, it will become very convenient to assess the functional expression of NP BGCs after random mutagenesis, genetic manipulation or exposure to various cultivation conditions. In the future, TF-based biosensors will hold great promise for accelerating cell factory development for pharmaceutical production and the activation of silent BGCs for the discovery of novel compounds in actinobacteria (Kim et al., 2016; Zarins-Tutt et al., 2016; Sun et al., 2017).

## Multiplex Site-Specific Genome Engineering for NPs Overproduction

In the last three decades, due to their broad host specificities, SSR systems have been widely applied to strain improvements, combinatorial biosynthesis, and heterologous expression of the entire BGCs in *Streptomyces*, *Actinoplanes* and other industrial actinobacteria (Baltz, 2012; Stark, 2017; Merrick et al., 2018). To date, at least ten SSR systems have been identified in actinobacteria, which are derived from bacteriophage CBG73463, R4, SV1, TG1, VWB, ΦBT1, ΦC31, ΦJoe, ΦK38-1 or Φ1/6 (Baltz, 2012; Fogg et al., 2017; Li et al., 2017a; Kormanec et al., 2019). In particular, ΦBT1, ΦC31 and TG1 systems have been widely used for cell factory development via multi-copy amplification of target genes or BGCs (Baltz, 2012). For instance, production titers of the antibiotic goadsporin were increased by 2.3-fold by a step-by-step introduction of two extra copies of the goadsporin BGC based on ΦC31 and TG1 integration systems (Haginaka et al., 2014). However, this strategy requires repeated rounds of conjugal transfer and is limited by the number of selection markers. In 2011, Murakami and coworkers demonstrated that the ZouA-RsA/B-mediated recombination system could be used to achieve tandem amplification of the ACT gene cluster with 4–12 copies, resulting in a 20-fold increase in ACT production (Murakami et al., 2011). However, the engineered strains may be genetically unstable in the absence of antibiotic selection due to the presence of tandem amplification. In addition, the method requires the introduction of the ZouA-RsA/B system into both flanks of target BGCs in advance via two-round conjugal transfer before BGC amplification, which is time-consuming and labor-intensive. To address these limitations, our group has recently developed two novel enabling technologies for multi-locus chromosomal integration of target genes or BGCs, including MSGE and aMSGE (Li et al., 2017b, 2019b).

### MSGE: Multiplex Site-Specific Genome Engineering Based on the “One Integrase-Multiple *attB* Sites” Concept

**FIGURE 2:**
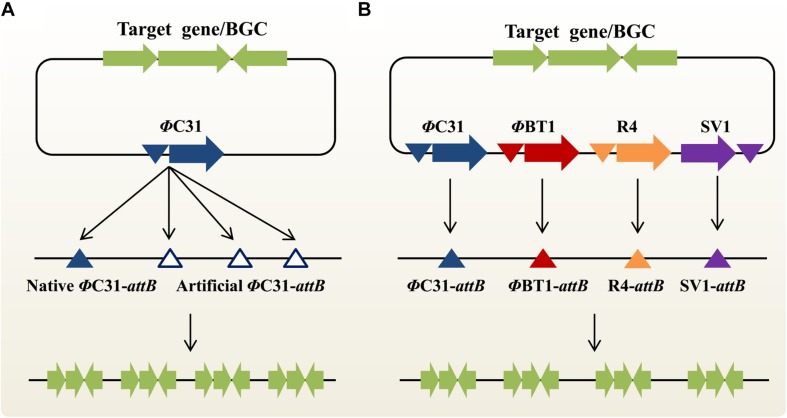
Two emerging synthetic biology approaches for multi-copy integration of target genes or natural products BGCs in actinobacteria. **(A)** Multiplex site-specific genome engineering (MSGE) for discrete amplification of target genes or BGCs. This method is based on the “one integrase-multiple *attB* sites” concept. The blue and blank triangles represent the native and artificial ΦC31 *attB* sites, respectively. **(B)** Advanced multiplex site-specific genome engineering (aMSGE) for multi-locus chromosomal integration of target genes or BGCs. This method is based on the “multiple integrases-multiple *attB* sites” concept. Particularly, these discrete *attB* sites are naturally occurring in the actinomycetal genomes. BGC, biosynthetic gene cluster.

In the actinomycetal genome, there is typically no, or only one, native *attB* site for each SSR system (Baltz, 2012). In theory, one-step, multi-copy integration of target genes or BGCs could be achieved by introducing multiple artificial *attB* sites into the host chromosome in advance. Based on this “one integrase-multiple *attB* sites” concept, an innovative approach MSGE was developed for discrete BGC amplification (Figure 2A; Li et al., 2017b). Using the high-efficiency CRISPR/Cas9 genome editing method (Huang et al., 2015), we sequentially introduced three ΦC31 and two ΦBT1 *attB* sites into the genomic loci of deleted, non-target secondary metabolite BGCs in the industrial strain *Streptomyces pristinaespiralis*. Then, five extra copies of the pristinamycin II BGC were integrated into the modified chromosome in two steps using the ΦC31 and ΦBT1 compatible integration systems, which led to significantly improved PII titers (Li et al., 2015, 2017b). Importantly, the novel strategy was also extended to develop a series of powerful *Streptomyces coelicolor* heterologous expression hosts. Up to four copies of the chloramphenicol or YM-216391 BGCs were simultaneously integrated into these new chassis strains, thus resulting in increased production titers (Li et al., 2017b). Using the highly effective heterologous expression system, YM-216391 BGC was engineered to generate aurantizolicin and a hybrid compound 3, which exhibits significantly increased antitumor activity (Pei et al., 2018). Similarly, Myronovskyi et al. (2018) also constructed a panel of cluster-free, powerful *Streptomyces albus* chassis strains based on the “one integrase-multiple *attB* sites” concept. The production titers of a variety of bioactive compounds, including aloesaponarin II, cinnamycin, didesmethylmensacarcin and griseorhodin A, were significantly enhanced in these heterologous expression superhosts (Myronovskyi et al., 2018). It is worth noting that compared with the ZouA-RsA/B recombination system, MSGE-based engineered strains would be genetically stable in the absence of antibiotic selection due to discrete, site-specific integration of target BGCs (Li et al., 2017b). However, when the MSGE method is used to amplify target BGCs with multiple copies in a single step, there is an upper limit to the number of integrated BGCs. For example, we found that the chloramphenicol or YM-216391 BGCs could be simultaneously inserted into *S. coelicolor* with up to 4 copies. The possible reason is that the high-order amplification of BGCs or the accumulation of target products places an excess burden on bacterial growth (Li et al., 2017b). In the near future, we believe that these versatile *Streptomyces* hosts will greatly accelerate (meta)genomic mining and combinatorial biosynthesis studies for novel bioactive NP discovery (Li et al., 2017a; Liu et al., 2018; Zhang et al., 2019).

### aMSGE: Advanced Multiplex Site-Specific Genome Engineering With Orthogonal Modular Recombinases

Although the MSGE method could be widely used to construct a variety of suitable chassis organisms for NP discovery and overproduction, two major limitations remains to be addressed. On the one hand, it will be difficult or even impossible to initially insert foreign *attB* sites into the chromosomes of genetically intractable industrial actinobacteria due to the lack of available replicative plasmids and also their low homologous recombination capability, such as spinosad-producing *Saccharopolyspora spinosa* and epoxomicin-producing *Goodfellowiella coeruleoviolacea*. On the other hand, repeated introduction of multiple artificial *attB* sites is still time-consuming and labor-intensive, especially for the slow-growing actinobacteria. To overcome these bottlenecks, our group recently developed an advanced MSGE method (aMSGE) based on the “multiple integrases-multiple *attB* sites” concept (Figure 2B; Li et al., 2019b). In this improved method, native *attB* sites of different orthogonal SSR systems in the actinomycetal genome are simultaneously applied to multi-copy integration of target genes or BGCs, rather than introducing foreign *attB* sites into the host chromosome. Accordingly, a plug-and-play amplification toolkit was designed and constructed, which contains 27 modular recombination plasmids with single or multiple SSR systems. Using this innovative technique, we achieved a high-efficiency introduction of the 5-oxomilbemycin A3/A4 BGC into the parental strain *Streptomyces hygroscopicus* with up to four extra copies, thus resulting in a significant increase in the production titers of 5-oxomilbemycin A3/A4 (Li et al., 2019b). Compared with previously developed metabolic engineering tools, the aMSGE method doesn’t require the introduction of any genetic modifications before target gene or BGC amplification, which will considerably simplify and accelerate efforts to facilitate NP discovery and overproduction. The whole process for BGC amplification takes only ∼18 days for the construction of high-yield engineered strains (e.g., growth period is ∼6 days). More importantly, the aMSGE method could be applicable to genetically intractable actinobacteria for strain improvements. Given that SSR systems are widely distributed in a variety of microorganisms (Fogg et al., 2014; Stark, 2017), our newly developed methodology should be widely extended to establish more efficient industrial platforms for overproducing valuable chemicals and drugs. However, the aMSGE method could not be used for BGC amplification in actinobacteria without native *attB* sites.

## Rational Pathway Refactoring of NP Biosynthesis

**FIGURE 3:**
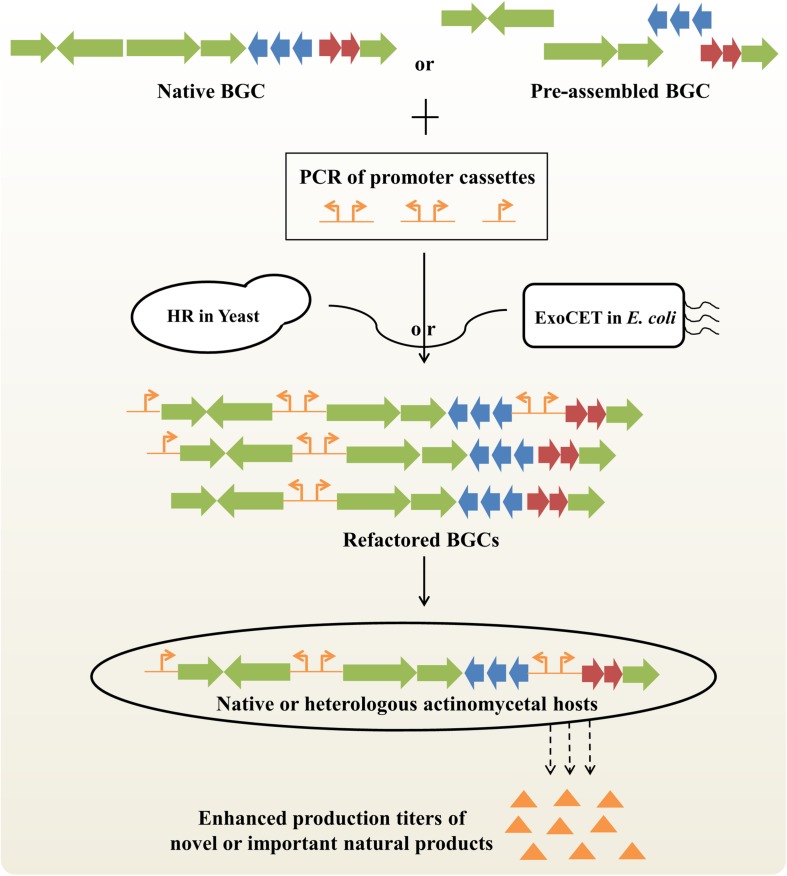
Improved production titers of novel or important natural products by BGC refactoring strategy. Native BGCs can be obtained by *in vivo* or *in vitro* BGC cloning/assembly strategies. Preassembled BGC can be obtained by PCR amplification or CRISPR-mediated *in vitro* digestion of native BGCs. Refactored BGCs can be obtained by partial or complete replacement of native promoters with artificial promoters based on homologous recombination (HR) in *S. cerevisiae* or exonucleases combined with RecET recombination (ExoCET) in *E. coli*. Finally, different refactored BGCs will be integrated into native or heterologous actinomycetal hosts for activating silent BGCs or enhancing production titers of clinically important drugs. BGC, biosynthetic gene cluster.

Generally, NP biosynthesis is under the control of highly complicated transcriptional, translational and metabolic regulation, which hampers the ability of systematic BGC engineering to maximize biosynthetic efficiency (Liu et al., 2013). Pathway refactoring provides an effective synthetic biological approach to decouple gene expression from complex native regulation and to achieve precise control of metabolite production by redesigning target BGCs in versatile surrogate hosts (Tan and Liu, 2017; Niu and Li, 2019). The key steps of BGC refactoring are to develop a set of well-characterized genetic control elements and high-efficiency DNA assembly methods (Figure 3).

In the last ten years, a series of synthetic regulatory elements have been identified to precisely regulate gene expression with wide dynamic ranges in actinobacteria, including constitutive or inducible promoters, ribosomal binding sites (RBSs), terminators and protein degradation tags (Myronovskyi and Luzhetskyy, 2016; Horball et al., 2018; Ji et al., 2019). For instance, the expression levels of 200 native or synthetic promoters and 200 artificial RBSs were systematically quantitated, which provides a universal toolbox of synthetic modular regulatory elements for the scalable and cost-effective optimization of NP biosynthetic pathway in different *Streptomyces* (Bai et al., 2015).

Next, a variety of *in vitro* or *in vivo* DNA assembly methods have been established to reconstruct target BGCs for diverse applications (Li et al., 2017a; Zhang et al., 2019). On the one hand, a panel of *in vivo* DNA assembly methods for single-marker or marker-free multiplexed promoter engineering of large BGCs have been developed on the basis of powerful homologous recombination capacity in *S. cerevisiae*, including DNA assembler (Shao et al., 2011), mCRISTAR (multiplexed Cas9-transformation-associated recombination) (Kang et al., 2016) and miCRISTAR (multiplexed *in vitro* Cas9-transformation-associated recombination) (Kim et al., 2019). For example, using the miCRISTAR strategy, the activation of a silent BGC led to the characterization of two bacterial cyclic sesterterpenes atolypene A and B (Kim et al., 2019). In another study, yeast-mediated construction of a riboswitch-controlled pathway achieved a 120-fold increase in bottromycin productivity (Eyles et al., 2018). Intriguingly, multiple new bottromycin-related metabolites were also generated by using high-efficiency, flexible BGC modifications. On the other hand, a range of *in vitro* DNA assembly methods suitable for pathway refactoring have been developed, including modified Gibson assembly (Li et al., 2015), MASTER (methylation-assisted tailorable ends rational) ligation (Chen et al., 2013) and SLIC (Sequence- and Ligation-Independent Cloning) (D’Agostino and Gulder, 2018). Recently, an innovative DNA assembly method, ExoCET (Exonuclease Combined with RecET recombination), was also developed for large-size, multi-operon BGC refactoring (Song et al., 2018; Wang et al., 2018). The artificial 79-kb spinosad BGC with 7 artificial operons under the control of strong constitutive promoters achieved a 328-fold enhanced spinosad production compared to the native BGC (Song et al., 2018). As a simple and robust genetic platform, BGC refactoring will have potentially broad applications in combinatorial biosynthesis and antibiotic overproduction, as well as high-throughput activation of silent BGCs from either metagenomes or uncultured actinobacteria.

## Construction of Genome-Reduced *Streptomyces* Hosts for NPs Discovery and Overproduction

*Streptomyces* species are capable of producing a wide range of secondary metabolites, such as polyketides, non-ribosomal peptides and terpenes, and possess the unique metabolic background needed for heterologous expression of NP BGCs from actinobacteria or other bacteria with high-GC content (Liu et al., 2018; Nepal and Wang, 2019). Within the streptomycetes, *S. coelicolor*, *S. albus* J1074 and *Streptomyces lividans* have been widely used as surrogate hosts for NPs discovery and overproduction (Beites and Mendes, 2015; Liu et al., 2018). With the continuous advances of novel bioinformatics tools and genetic manipulation techniques (Zhang et al., 2016, 2019; Ziemert et al., 2016), a series of versatile *Streptomyces* chassis have been developed by deleting non-essential genomic regions, such as redundant BGCs, genome islands and insertion sequences (Liu et al., 2018; Huo et al., 2019; Table 1). Particularly, deletion of redundant BGCs are predicted to increase the supply of primary metabolite precursors, remove competing carbon and nitrogen sinks, and also facilitate the biosynthesis of heterologous BGCs. In addition, engineered strains without redundant BGCs will possess simple extracellular metabolite profiles for convenient structural characterization of novel bioactive compounds.

**TABLE 1:** Characteristics of synthetic model *Streptomyces* chassis.

**Model**	***Streptomyces coelicolor***			***Streptomyces albus***		***Streptomyces lividans***
**strains**	**M145**			**J1074**		**TK24**
Engineered hosts	M1146/M1152	M1246-M1446/M1252-M1452	M1317	Del14	B2P1/B4	SBT5
Characteristics	Deletion of BGCs for ACT, CDA, CPK and RED	Derived from M1146 or M1152 with 1-3 artificial ΦC31 *attB* sites	Derived from M1152 by deleting all three of type III polyketide genes	Deletion of 15 endogenous BGCs	Derived from Del14 with 1-2 artificial ΦC31 *attB* sites	Deletion of BGCs for ACT, RED and CDA and insertion of *afsRS*_cla_
Deletion sizes	173 kb (2%)	173 kb (2%)	176 kb (2%)	500 kb (7.3%)	500 kb (7.3%)	120 kb (1.4%)
Deletion methods	Resistance gene- assisted recombination	CRISPR-based recombination	RedET-mediated recombination	RedET-mediated recombination	RedET-mediated recombination	Resistance gene- assisted recombination
Compounds	Chaxamycin, Taromycin B, Thiostreptamide et al.	Chloramphenicol and YM-216391	Flaviolin and Germicidin	Cinnamycin, Griseorhodine A, Tunicamycin B2 et al.	Cinnamycin, Griseorhodine A, Tunicamycin B2 et al.	8D1-1 and 8D1-2
Reference	Gomez-Escribano and Bibb, 2011	Li et al., 2017b	Thanapipatsiri et al., 2015	Myronovskyi et al., 2018	Myronovskyi et al., 2018	Bai et al., 2014

*ACT, actinorhodin; CDA, calcium-dependent antibiotic; CPK, cryptic type I polyketide; RED, prodiginine; BGC, biosynthetic gene cluster.*

Along with the emergence of genome mining as a robust approach to discover novel drug leads, the development of reliable *Streptomyces* chassis for heterologous expression of cloned BGCs is becoming increasingly important (Huo et al., 2019; Nepal and Wang, 2019). As a model strain, *S. coelicolor* has been widely used to study the molecular regulation of antibiotic biosynthesis and morphological differentiation (Liu et al., 2013; Barka et al., 2016). In the last 10 years, a series of advanced genome editing tools and diverse synthetic regulatory elements have been developed for the optimization of heterologous pathways in *S. coeliclor* (Myronovskyi and Luzhetskyy, 2016; Alberti and Corre, 2019). In 2011, on the basis of the wild-type strain *S. coelicolor* M145, two versatile engineered hosts M1146 and M1152 were developed, which contained deletions in four BGCs, responsible for the biosynthesis of ACT, calcium-dependent antibiotic (CDA), cryptic type I polyketides (CPK) and prodiginine (RED), or in combination with a point mutation in *rpoB* (Gomez-Escribano and Bibb, 2011). The productivity of the chloramphenicol or congocidine BGCs was significantly increased compared with the parental strain (Gomez-Escribano and Bibb, 2011). Nowadays, M1146 and M1152 have been widely used to heterologously express different types of compounds from fastidious original producers or metagenomic DNA (Huo et al., 2019; Nepal and Wang, 2019). Similarly, a genome-reduced host *S. lividans* SBT5 was also developed by deleting BGCs responsible for ACT, CDA and RED as well as inserting the regulatory gene *afsRS*_cla_ (Bai et al., 2014). Furthermore, to specifically facilitate the study of Type III polyketides, an effective surrogate *S. coelicolor* host M1317 was constructed by removing all three endogenous Type III polyketide synthase (PKS) genes (*gcs*, *srsA*, *rppA*) on the basis of M1152, which could potentially increase precursor supply and prevent undesirable interference with expression of heterologous Type III PKS genes (Thanapipatsiri et al., 2015). As another important *Streptomyces* chassis, *S. albus* J1074 provides high success rates of heterologous BGC expression with rapid growth and high productivity (Nepal and Wang, 2019). Recently, on the basis of *S. ablus* J1074, 15 endogenous secondary metabolite BGCs were deleted to generate a powerful engineered host Del14 (Myronovskyi et al., 2018). The production yields of heterologously expressed metabolites in *S. albus* Del14 were higher than those in commonly used *S. albus* J1074 and *S. coelicolor* M1152. Furthermore, the authors also introduced 1-2 artificial ΦC31 *attB* sites into the genomes of *S. albus* Del14, thus resulting in two another versatile engineered hosts, B2P1 and B4. The two powerful chassis could be easily used for BGC amplification of up to four copies, thus efficiently improving fermentation levels of the encoded compounds (Myronovskyi et al., 2018).

In addition to the construction of a model heterologous expression chassis, the genome-reduced strategy has been frequently used to develop non-model *Streptomyces* strains for the discovery and overproduction of important NPs (Table 2). *Streptomyces* sp. FR-008 is a fast-growing, potentially industrial production chassis, which produces the macrolide candicidin and other bioactive compounds. By deleting three endogenous polyketide genes, a mutant strain LQ3 was constructed with a stable and streamlined genome structure, which possibly allows for simple separation and purification of heterologously expressed compounds (Liu et al., 2016). As a producer of the anti-infective avermectin, *Streptomyces avermitilis* is already optimized to efficiently supply primary precursors. To construct a versatile industrial chassis for heterologous expression of secondary metabolite BGCs, a series of genome-reduced *S. avermitilis* mutants were obtained by deleting the left subtelomeric region (∼2 Mb) that corresponds to the more variable genome regions (Komatsua et al., 2010). Herein, the SUKA5 and SUKA17 strains were highlighted, which had genome reductions of 17.9 and 18.5%, respectively. *S. avermitilis* SUKA5 strain has the deletions of both oligomycin BGC and the left subtelomeric region, and the SUKA17 strain is a derivative of SUKA5 with the additional deletions of three terpene BGCs (Komatsua et al., 2010). The feasibility and superiority of these two engineered hosts has been widely confirmed by the efficient production of more than 20 exogenous secondary metabolite BGCs (Komatsu et al., 2013; Ikeda et al., 2014). Similarly, 1.3-Mb and 0.7-Mb possible non-essential genomic regions were deleted in the natamycin-producing industrial strian *Streptomyces chattanoogensis* L10 by Cre-loxP recombination system, respectively, thus generating two efficient chassis L320 and L321 for the production of valuable polyketides (Bu et al., 2019). In another study, a newly engineered host ZXJ-6 was developed based on a salinomycin-producing industrial strain, *Streptomyces albus* BK3-25, by deleting the salinomycin BGC and chromosomal integration of a three-gene cassette for the biosynthesis of ethylmalonyl-CoA. It was successfully used for the heterologous expression of polyketide BGCs, such as the ACT BGC from *S. coelicolor* (Zhang et al., 2017). In the future, the genome-reduced strategy could be widely implemented in a variety of industrial actinobacteria for the improved production of various bioactive compounds.

**TABLE 2:** Characteristics of synthetic non-model *Streptomyces* chassis.

**Non-model**	***Streptomyces* sp.**	***Streptomyces avermitilis***		***Streptomyces albus***	***Streptomyces chattanoogensis***	
**strain**	**FR-008**	**wide-type**		**BK3-25**	**L10**	
Engineered host	LQ3	SUKA5	SUKA17	ZXJ-6	L320	L321
Characteristic	Deletion of BGCs for three endogenous polyketide genes	Deletion of left subtelomeric region and oligomycin BGC	Derived from SUKA5 and deletion of BGCs for three terpene compounds	Introduction of the ethylmalonyl-CoA biosynthetic pathway and deletion of the salinomycin BGC	Deletion of possible non-essential 0.5–1.8 Mb genomic region	Deletion of possible non-essential 8–8.7 Mb genomic region
Deletion size	150 kb (2.1%)	1.62 Mb (17.9%)	1.67 Mb (18.5%)	77 kb (0.9%)	1.3 Mb (14.4%)	0.7 Mb (7.8%)
Deletion method	Resistance gene- assisted recombination	Cre-loxP recombination	Cre-loxP recombination	Resistance gene-assisted recombination	Cre-loxP recombination	Cre-loxP recombination
Compound	NA	Cephamycin C, Pladienolide, Streptomycin et al.	Kasugamycin, Oxytetracycline, Rebeccamycin et al.	ACT	Natamycin	Natamycin
Reference	Liu et al., 2016	Komatsua et al., 2010	Komatsua et al., 2010	Zhang et al., 2017	Bu et al., 2019	Bu et al., 2019

*ACT, actinorhodin; BGC, biosynthetic gene cluster; PKS, polyketide synthase gene; NRPS, non-ribosomal peptide synthase gene; NA, not available.*

## Concluding Remarks

Actinobacteria, especially bacteria from the genus *Streptomyces*, have long been employed as an important source of a wide range of novel bioactive small molecules (Barka et al., 2016; Newman and Cragg, 2016; Genilloud, 2017). To harness the production potential of actinobacteria, a variety of innovative metabolic engineering and synthetic biology strategies have been developed in the last 10 years, including dynamic metabolic regulation, BGC amplification, pathway refactoring and genome-minimized *Streptomyces* chassis (Beites and Mendes, 2015; Tan and Liu, 2017; Tan and Prather, 2017; Li et al., 2019a). We envision that NP discovery and development will be rapidly accelerated by the refactoring and amplification of whole biosynthetic pathways in combination with powerful heterologous expression platforms. Considering that a large number of BGCs are generally silent or expressed at very low levels, it is still important to develop diverse heterologous hosts and universal refactoring approaches to activate silent BGCs or boost production of secondary metabolites. Next, quorum sensing systems are widely distributed in actinobacteria (Polkade et al., 2016), which provide opportunities for bacterial growth-mediated dynamic regulation of metabolic pathways for enhanced production of target compounds. Furthermore, due to the complex regulation between primary and secondary metabolism, iterative application of the design-build-test-learn cycle will be necessary to overproduce different secondary metabolites (Liu et al., 2013; Kim et al., 2016; van der Heul et al., 2018). Finally, the advancements in the fields of genome sequencing, multi-omics and genome editing techniques are paving the way for systems metabolic engineering of industrial actinobacteria, including pathway engineering, regulatory circuit rewiring, host modification and enzyme engineering (Kim et al., 2016; Zhang et al., 2019). Given the requirement for high titers in commercial production, an integrated approach involving traditional mutagenesis screening and rational host/pathway engineering is required to systematically optimize the biosynthesis of target compounds. It is expected that these synthetic biology tools and metabolic engineering strategies presented in this review and future developments will play increasingly important roles in the discovery of novel drug leads, as well as yield improvement for large-scale manufacturing in actinobacteria.

## Author Contributions

LL and XL wrote the manuscript. LL, WJ, and YL reviewed, edited, and approved its final version.

## Conflict of Interest

The authors declare that the research was conducted in the absence of any commercial or financial relationships that could be construed as a potential conflict of interest.
